# Immunobiotic *Lactobacillus rhamnosus* strains differentially modulate antiviral immune response in porcine intestinal epithelial and antigen presenting cells

**DOI:** 10.1186/1471-2180-14-126

**Published:** 2014-05-16

**Authors:** Julio Villena, Eriko Chiba, Maria Guadalupe Vizoso-Pinto, Yohsuke Tomosada, Takuya Takahashi, Takamasa Ishizuka, Hisashi Aso, Susana Salva, Susana Alvarez, Haruki Kitazawa

**Affiliations:** 1 Food and Feed Immunology Group, Laboratory of Animal Products Chemistry, Graduate School of Agricultural Science, Tohoku University, Sendai 981-8555, Japan; 2 Laboratory of Immunobiotechnology, Reference Centre for Lactobacilli (CERELA-CONICET), Tucuman, Argentina; 3 INSIBIO-CONICET, Biomedical Department, Faculty of Medicine, National University of Tucumán, Tucumán, Argentina; 4 Cell Biology Laboratory, Graduate School of Agricultural Science, Tohoku University, Sendai 981-8555, Japan

**Keywords:** *Lactobacillus rhamnosus*, Poly(I:C), Antiviral immunity, PIE cells, Intestinal antigen presenting cells, TLR2

## Abstract

**Background:**

Previous findings suggested that *Lactobacillus rhamnosus* CRL1505 is able to increase resistance of children to intestinal viral infections. However, the intestinal cells, cytokines and receptors involved in the immunoregulatory effect of this probiotic strain have not been fully characterized.

**Results:**

We aimed to gain insight into the mechanisms involved in the immunomodulatory effect of the CRL1505 strain and therefore evaluated in vitro the crosstalk between *L. rhamnosus* CRL1505, porcine intestinal epithelial cells (IECs) and antigen presenting cells (APCs) from swine Peyer’s patches in order to deepen our knowledge about the mechanisms, through which this strain may help preventing viral diarrhoea episodes. *L. rhamnosus* CRL1505 was able to induce IFN–α and –β in IECs and improve the production of type I IFNs in response to poly(I:C) challenge independently of Toll-like receptor (TLR)-2 or TLR9 signalling. In addition, the CRL1505 strain induced mRNA expression of IL-6 and TNF-α via TLR2 in IECs. Furthermore, the strain significantly increased surface molecules expression and cytokine production in intestinal APCs. The improved Th1 response induced by *L. rhamnosus* CRL1505 was triggered by TLR2 signalling and included augmented expression of MHC-II and co-stimulatory molecules and expression of IL-1β, IL-6, and IFN-γ in APCs. IL-10 was also significantly up-regulated by CRL1505 in APCs.

**Conclusions:**

It was recently reviewed the emergence of TLR agonists as new ways to transform antiviral treatments by introducing panviral therapeutics with less adverse effects than IFN therapies. The use of *L. rhamnosus* CRL1505 as modulator of innate immunity and inductor of antiviral type I IFNs, IFN-γ, and regulatory IL-10 clearly offers the potential to overcome this challenge.

## Background

Around 5.2 million children under five years old die yearly due to preventable infectious diseases like pneumonia and diarrhoea [1,2]. Among these infectious diseases, viral gastrointestinal infections belong to the most frequent diseases suffered in childhood, especially in the developing world. Rotavirus, a RNA virus, is the most common cause of severe dehydrating diarrhoea in children worldwide [3,4]. Although there is already a successful rotavirus vaccine in the market, the epidemic in the developing world is far from being controlled [4,5]. Apart from being not affordable for low-income population groups, it has also been shown that protection induced by natural infection and vaccination is reduced in developing areas, where among other factors, children are infected at an early age and high viral challenge loads are usual [6]. Moreover, Latin America in general and northern Argentina in particular, presents a significant population of malnourished children with its associated burden of otherwise preventable infectious diseases such as rotavirus infections [2].

Several studies have demonstrated that certain lactic acid bacteria (LAB) strains can exert their beneficial effect on the host through their immunomodulatory activity. In this sense, some studies have centred on whether immunoregulatory probiotic LAB (immunobiotics) might sufficiently stimulate the intestinal immune system to provide protection against viral infections. It was reported that probiotics can exerts some beneficial effects in rotavirus intestinal infections such as shortening the duration of diarrhoea, reducing the number of episodes, lessening rotavirus shedding, normalizing gut permeability and increasing the production of rotavirus-specific antibodies [7-9]. In an attempt to find low-cost alternatives for the prevention of infectious diseases we have developed a new probiotic yogurt, containing the immunobiotic strain *Lactobacillus rhamnosus* CRL1505, able to improve resistance against respiratory and intestinal infections. Our studies in animal models showed that the administration of *L. rhamnosus* CRL1505 significantly augmented the resistance of immunocompetent and immunocompromised malnourished mice to intestinal and respiratory pathogens such as S*almonella* Typhimurium and *Streptococcus pneumoniae*[10,11]. In addition, we performed a randomized controlled trial in order to evaluate the effect of the probiotic yogurt containing *L. rhamnosus* CRL1505 on both gut and non-gut related illnesses among children [12]. We demonstrated that the CRL1505 strain improved mucosal immunity and reduced the incidence and severity of intestinal and respiratory infections. We registered that 34% of the children who consumed the probiotic yogurt showed some type of infectious event, while in the placebo group this value was higher reaching a 66% of them. Although we did not evaluate aetiology of intestinal and respiratory infections in the clinical study, previous evaluations have shown that viruses, such as rotavirus and respiratory syncytial virus, are the major pathogens, which cause infectious diseases in children in northern Argentina [13,14]. Therefore, our findings suggested that administration of *L. rhamnosus* CRL1505 may provide a potential intervention to prevent the course of common childhood viral infections. Some of the mechanisms by which *L. rhamnosus* CRL1505 exerts its immunomodulatory and antiviral properties have been elucidated [10,11,15]. We have recently showed the capacity of the CRL1505 strain to improve the production of antiviral cytokines in the gut and the respiratory tract [10,11,15,16]. However, the intestinal cells, cytokines and receptors involved in the immunoregulatory effect of this immunobiotic strain have not been fully characterized.

Intestinal epithelial cells (IECs) are the first cells which encounter exogenous and endogenous as well as pathogenic and non-pathogenic microorganisms [17]. In addition, the gut of vertebrates is rich in antigen-presenting cells (APCs), such as macrophages and dendritic cells (DCs), which are able to recognize foreign antigens or invading pathogens. The epithelium and APCs at the intestinal surfaces express a diverse range of Pattern Recognition Receptors (PRRs) capable of detecting viruses. Epithelial- and APCs-expressed PRRs include cell surface expressed C-type lectins (cell surface variants of the secreted collectins), intra- and extracellular toll-like receptors (TLR), the intracellular RNA-dependent protein kinase (PKR), retinoic acid–inducible gene I (RIG-I) like receptors (RLR) and nucleotide binding domain and leucine-rich repeat containing receptors (NLR) [18-20]. Upon recognition of double-stranded RNA (dsRNA) or its synthetic analogue poly(I:C), TLR3 and RIG-I trigger the activation of the transcription factors IRF-3, NF-kB, and AP-1, which in turn induce type I IFNs (especially IFN-β) and cytokine/chemokine synthesis.

There is a growing interest in studying the swine immune system because of its similarities to the human immune system. We have precisely characterized the functionality of porcine APCs from Peyer’s Patches (PPs) before and also demonstrated that swine PPs-derived adherent cells are a useful *in vitro* tool for investigating innate responses to pathogenic and probiotic microorganisms [21]. In addition, we have also reported an abundant intracellular expression of TLR3 in a porcine intestinal epithelial (PIE) cell line [22], which is in line with findings of Liu et al. [8] that demonstrated that the non-transformed porcine jejunum epithelial cell line (IPEC-J2) expresses TLR3 constitutively. We characterized the immune response triggered by poly(I:C) challenge in PIE cells and in PIE-immune cell co-cultures, and demonstrated that these systems are valuable tools for studying *in vitro* the immune response triggered by TLR3/RIG-I on IECs and the interaction between IECs and immune cells [22,23]. In this study, we therefore aimed to use these porcine *in vitro* systems to gain insight into the mechanisms involved in the immunomodulatory effect of CRL1505 strain, and concentrated our attention in the crosstalk between *L. rhamnosus* CRL1505, PIE cells and APCs in order to deepen our knowledge about the mechanisms, through which this strain may help preventing viral diarrhoea episodes.

## Methods

### Microorganisms

*Lactobacillus rhamnosus* CRL1505 (Lr1505) and *L. rhamnosus* CRL1506 (Lr1506) belong to CERELA Culture Collection and were originally isolated from goat milk [11]. These strains were grown in Man-Rogosa-Sharpe (MRS) broth at 37°C. For immunomodulatory assays, overnight cultures were harvested by centrifugation, washed three times with sterile PBS, counted in a Petroff-Hausser counting chamber, and re-suspended in DMEM until use.

### PIE cell monocultures

A non-transformed porcine intestinal epithelial cell line (PIE), characterized by its ability to build a monolayer with a cobblestone and epithelial-like morphology and close contacts between cells was used as described before [22,23]. Briefly, PIE cells were grown on type I collagen-coated dishes using DMEM (Gibco, Japan) supplemented with 10% fetal calb serum (FCS, Sigma). PIE cells were incubated at 37°C and 5% CO_2_. Passages were done by treating the monolayer with sucrose/EDTA for 4 min and detaching the cells with 0.04% trypsin.

### Isolation of adherent population from swine Peyer’s patches (PPs)

Suspensions of porcine PP immunocompetent cells were prepared from adult swine intestine. This study was carried out in strict accordance with the recommendations in the Guide for the Care and Use of Laboratory Animals of the Guidelines for Animal Experimentation of Tohoku University, Sendai, Japan. The present study was approved by the Institution Animal Care and Use Committee of Tohoku University with a permitted No. 2011-noudou-5 and all efforts were made to minimize suffering. Swine PPs were cut into small pieces and gently pressed through a nylon mesh to prepare single immune cell suspensions. After several washes in complete RPMI medium, residual erythrocytes were lysed in 0.2% NaCl followed by a hypertonic rescue in 1.5% NaCl. Finally, immune cells were fractioned by density gradient centrifugation using Lympholyte Mammal (Cedarlane, Corby, Canada) and the mononuclear cell suspension containing a mixed population of T, B and antigen presenting cells (APCs) was suspended in complete DMEM supplemented with 10% FCS, 50 μg/ml penicillin/streptomycin and 50 μg/ml gentamycin (Nacalai Tesque, Kyoto, Japan) [22,23]. APCs (macrophages and DCs) were separated by their ability to adhere to glass as described before [21]. Briefly, cell suspensions (5 × 10^7^ cells/well) were placed onto 2-well glass plates (Iwaki, Tokyo, Japan) and incubated for 2 h at 37°C and 5% CO_2_ to allow cells to adhere to the glass surface. Subsequently, they were washed gently with complete RPMI 1640 medium (Sigma) to remove non-adherent cells. With this methodology a mix population containing CD172a^+^CD11R1^−^, CD172a^−^CD11R1^low^ and CD172a^+^CD11R1^high^ cells was obtained [21].

### Immunomodulatory effect of lactobacilli

Evaluation of the immunomodulatory activity of *L. rhamnosus* CRL1505 and *L. rhamnosus* CRL1506 was performed using PIE cells and PPs-derived adherent cells [21-23]. For immunomodulatory assays, 1.5 × 10^4^ PIE cells/well were plated onto type I collagen coated 24-well plates (Iwaki, Tokyo, Japan). Three days later, cell monolayers were washed, added with lactobacilli (5 × 10^8^ cells/well) and incubated for 48 h at 37°C and 5% CO_2_, after which cells were vigorously washed and harvested for total RNA isolation for cytokine expression profiles. In a second experiment to study immunomodulation of antiviral innate responses with lactobacilli, PIE cell monolayers were incubated 48 h with lactobacilli, washed three times to eliminate possible stimulants and were further stimulated with poly(I:C) to mimic viral infection at the indicated times. Again, RNA was isolated for studying expression profiles [22,23]. Adherent cells were plated at a density of 1.5 × 10^6^ cells/well in 12-well type I collagen-coated plates (Iwaki) or in 2-well glass plates (Iwaki). Lactobacilli were added to each well (5 × 10^8^ cells/ml) and incubated for further 16 h. For evaluation of the modulation of antiviral responses by lactobacilli in APCs, adherent cells were prepared as indicated before and 16 h later, each well was washed vigorously with medium at least 3 times to eliminate bacteria; and finally the porcine cells were stimulated with poly(I:C) for the time indicated [21]. In addition, unlabelled anti-TLR2 rabbit IgG or anti-TLR9 rabbit IgG (Santa Cruz, Santa Cruz, CA) were used in blocking experiments. Cultured cells were incubated with the unlabelled anti-TLR2 or anti-TLR9 antibodies for 12 h before stimulation with lactobacilli.

### Lactobacilli immunomodulatory activity in PIE-adherent cells co-culture system

Porcine PPs adherent cells suspensions were prepared as described above. In the Transwell culture system, PIE cells were seeded in the apical surface at a concentration of 1.5 × 10^5^ cells/well in 12-well tissue culture plates (Transwell-Col. (PTFE), pore size 0.2 mm) while porcine PPs adherent cells were seeded in the basolateral compartment at a concentration of 2 × 10^7^ cells/well [22,23]. For the evaluation of the immunomodulatory activity of lactobacilli in the PIE-immune cell co-culture system, the apical surface containing PIE cells was stimulated with lactobacilli strains for 48 h and then washed twice with PBS. Finally, PIE cells were stimulated with poly(I:C) for 12 h.

### qRT-PCR of mRNA expression in PIE and immune cells

Total RNA from each stimulated monolayer (PIE cell monoculture or co-culture) was isolated using TRIzol reagent (Invitrogen) according to the manufacturer’s instructions. cDNA was synthesized using a Quantitect Reverse Transcription kit (Qiagen, Tokyo, Japan). qRT-PCR was carried out in a 7300 Real-time PCR System (Applied Biosystems, Warrington, Cheshire, UK) using Platinum SYBR Green qPCR SuperMix UDG with ROX (Invitrogen). The primers for IFN-α, IFN-β, TNF-α, IFN-γ, IL-1β, TGF-β, IL-2, IL-6, IL-10 and IL-12p40 used in this study were described previously [24]. The PCR cycling conditions were 5 min at 50°C; followed by 2 min at 95°C; then 40 cycles of 15 sec at 95°C, 30 sec at 60°C and 30 sec at 72°C. The reaction mixture contained 5 μl cDNA and 15 μl master mix including sense and antisense primers. Expression of the house-keeping gene b-actin was assessed in each sample, as an internal control to normalize differences between samples and to calculate the relative index.

### Flow cytometric analysis

Flow cytometry was used to assess expression of MHC-II, CD80/86, IFN-γ, IL-1β, IL-6 and IL-10 in PPs CD172a^+^CD11R1^−^, CD172a^−^CD11R1^low^ and CD172a^+^CD11R1^high^ cells. Adherent cells were isolated as described above and labeled with primary antibodies: anti-porcine CD172a-PE SWC3 IgG1 (Southern Biotech), anti-porcine CD11R1-IgG1 (AbD Serotec), anti-porcine MHC-II-IgG2a (VMRD), anti-porcine gamma interferon (IFN-γ)-IgG2b (R&D Systems, Minneapolis, MN), anti-porcine interleukin-10 (IL-10)-IgG2b (R&D Systems), anti-porcine IL-1β/IL-1 F2-IgG1 (R&D Systems), and anti-porcine IL-6-IgG2b (R&D Systems). The binding of unlabeled monoclonal antibodies was visualized using the following secondary antibodies: anti-mouse IgG1-peridinin chlorophyll protein (PerCP)/Cy5.5 (Bio Legend, San Diego, CA), anti-mouse IgG2a-FITC (AbD Serotec), anti-rabbit IgG-Alexa Fluor 489 (Santa Cruz), anti-mouse IgG2b-FITC (AbD Serotec), and anti-mouse IgG-FITC (AbD Serotec) [21]. In addition, expression levels of CD80/86 proteins were evaluated using a human CD152 (cytotoxic-T- lymphocyte-associated antigen 4) Ig/FITC fusion protein (Ancell, Bay- port, MN). Cells stained with irrelevant mouse IgG-FITC, IgG2b-FITC, IgG2a-PerCP, IgG2b-PE, IgG2a-PE, or IgG1-PE antibodies (eBioscience, San Diego, CA) were included as isotype controls. Analysis of the stained cells was performed using a FACSCalibur flow cytometer (BD, Franklin Lakes, NJ), which was equipped with Cell-Quest software. Data analysis was performed using FlowJo software (Tree Star, Ashland, OR) [21].

### Statistical analysis

Statistical analyses were performed using the GLM and REG procedures available in the SAS computer program (SAS, 1994). Comparisons between mean values were carried out using one-way analysis of variance and Fisher’s least-significant-difference (LSD) test. *P* < 0.05 were considered significant.

## Results

### *Lactobacillus rhamnosus* strains differentially modulate cytokines transcriptional profiles of PIE cells and PPs derived adherent cells

**Figure 1:**
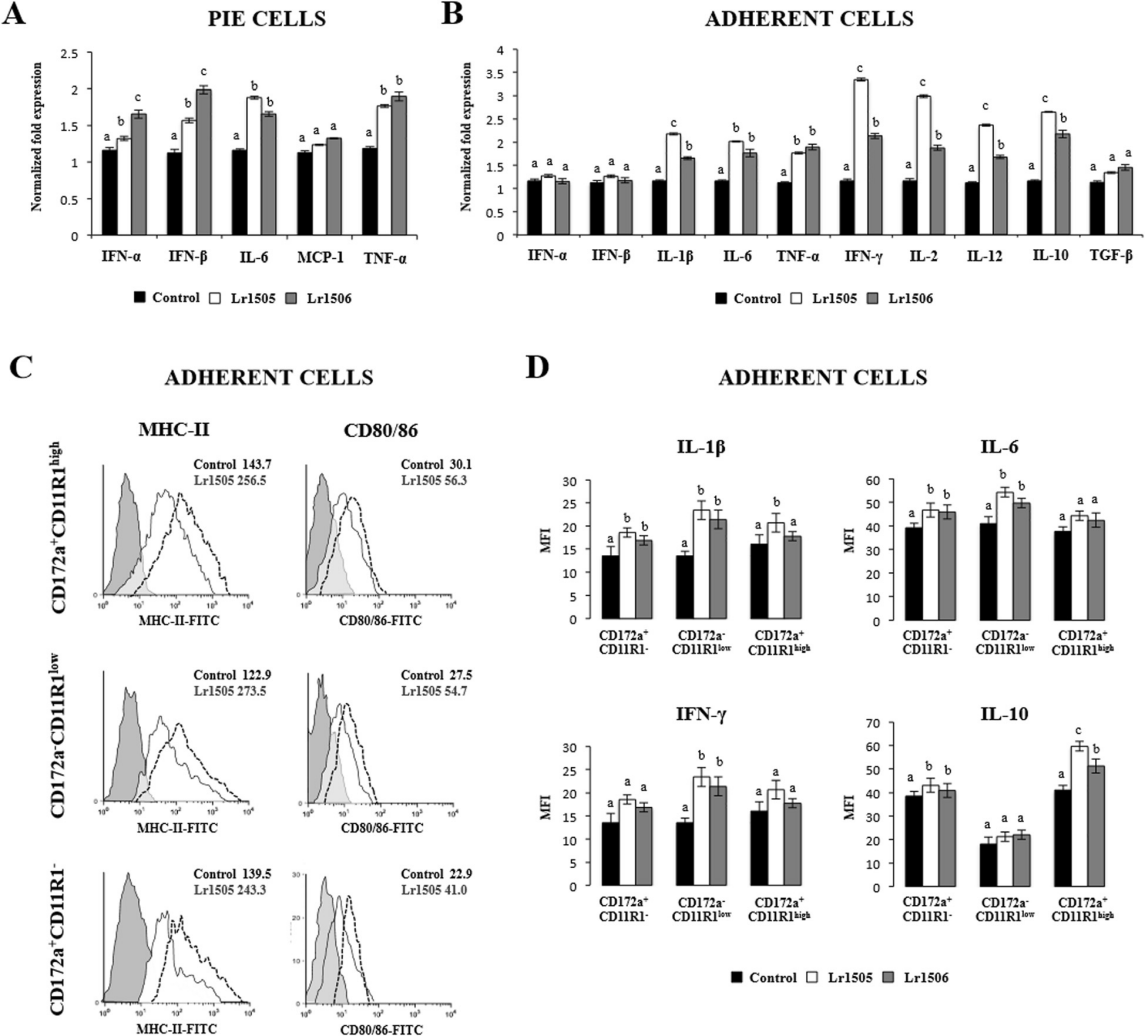
**Effect of immunobiotic lactobacilli in porcine intestinal epithelial (PIE) cells and antigen presenting cells (APCs) from Peyer’s patches.** Monocultures of PIE cells or adherent cells from Peyer’s patches were stimulated with *Lactobacillus rhamnosus* CRL1505 (Lr1505) or *L. rhamnosus* CRL1506 (Lr1506). The mRNA expression of IFN-α, IFN-β, IL-6, MCP-1 and TNF-α was studied in PIE cells after 48 hours of stimulation **(A)**. The mRNA expression of IFN-α, IFN-β, IL-1β, TNF-α, IFN-γ, IL-6, IL-2, IL-12, IL-10 and TGF-β was studied in adherent cells after 12 hours of stimulation **(B)**. Cytokine mRNA levels were calibrated by the swine β-actin level and normalized by common logarithmic transformation. In addition, expression of MHC-II and CD80/86 molecules **(C)** as well as intracellular levels of IL-1β, IL-10, IFN-γ and IL-10 **(D)** were studied in the three populations of APCs within adherent cells defined with CD172a and CD11R1 markers. Values represent means and error bars indicate the standard deviations. The results are means of 3 measures repeated 4 times with independent experiments. The mean differences among different superscripts letters were significant at the 5% level.

The first aim of this study was to evaluate the effect of Lr1505 on the cytokine mRNA expression profile of PIE cells and PPs adherent cells. In addition, we used a second strain, Lr1506, also isolated from goat milk, to comparatively evaluate their effects. Both lactobacilli have similar technological properties and the ability to improve intestinal immunity [11,16]. However, Lr1506 is not able to improve respiratory immunity when orally administered, therefore comparative studies with both Lr1505 and Lr1506 offer a unique opportunity to study the mechanisms involved in the immunoregulatory effects of probiotics. Hence, PIE cell monolayers were stimulated with Lr1505 or Lr1506 for 48 h and the expression of several cytokines was quantified by qRT-PCR (Figure 1A). The expression levels of mRNA coding for IFN-α, IFN-β, IL-6 and TNF-α were significantly increased by both lactobacilli strains (Figure 1A). Furthermore, while TNF-α and IL-6 mRNAs were up-regulated to similar levels by both strains, the up-regulation of both IFN-α and IFN-β by Lr1506 was significantly higher than those induced by Lr1505 (Figure 1A). In addition, MCP-1 mRNA expression remained unchanged for all treatments.

In a similar setting but using APCs, Lr1505 and Lr1506 also showed a differential effect on the mRNA expression of some cytokines as shown in Figure 1B. Although both strains stimulated adherent cells, Lr1505 showed a stronger enhancing influence than Lr1506 on the expression of mRNA coding for IL-1β, IFN-γ, IL-2, IL-12 and IL-10 (Figure 1B). Both lactobacilli slightly but significantly increased the mRNA synthesis of IL-6 and TNF-α to similar levels. In contrast to the results seen in PIE cells, there was no meaningful effect on the mRNA expression of type I IFN (Figure 1B). Furthermore, TGF-β mRNA levels were not affected by the stimulation with lactobacilli.

### *L. rhamnosus* CRL1505 and CRL1506 stimulate PPs APCs and distinctly modulate cytokine production

We next studied whether Lr1505 and Lr1506 were able to affect the expression of two cellular surface markers for APCs activation: MHC-II and CD80/CD86. Adherent cells isolated from swine Peyer’s Patches can be grouped as CD172a^+^CD11R1^high^, CD172a^−^CD11R1^low^ and CD172a^+^CD11R1^−^ cells [21]. Although more detailed functional studies are needed to accurately define each population, it has been suggested that CD172a^+^CD11R1^high^ and CD172a^−^CD11R1^low^ cells could be considered as DCs and CD172a^+^CD11R1^−^ cells could be considered as macrophages [21]. In these three cell populations, both strains exerted an up-regulation of the antigen presenting and co-stimulatory molecules MHC-II and CD80/86, when compared to the non-stimulated control (Figure 1C) indicating that these immunobiotic microorganisms were able to activate APCs. In all cases the MIF values in Lr1505-treated cells almost doubled the MIF presented by control cells (Figure 1C). APCs were similarly modulated by Lr1506 (data not shown). We also analysed by flow cytometry the levels of IL-1β, IL-6, IFN-γ, and IL-10 on the three populations of adherent cells: CD172a^+^CD11R1^−^, CD172a^−^CD11R1^low^ and CD172a^+^CD11R1^high^ (Figure 1D). In CD172a^+^CD11R1^−^ cells both strains Lr1505 and Lr1506 slightly but significantly enhanced the post-translational expression levels of IL-1β, IL-6, and IL-10, while the IFN-γ levels remained unchanged (Figure 1D). In CD172a^−^CD11R1^low^ cells, both strains had a similar effect on the expression of IL-1β, IL-6 and IFN-γ, whereas IL-10 levels were not modified. In contrast, in CD172a^−^CD11R1^high^ cells IL-10 protein levels were up-regulated by both strains, being Lr1505 the strain which showed the strongest stimulation (Figure 1D). In addition, IL-1β was modulated only by Lr1505 but neither IL-6 nor IFN-γ levels were affected by the stimulation of CD172a^−^CD11R1^high^ cells with lactobacilli (Figure 1D). These results correlated with the mRNA expression profiles shown before (Figure 1B).

### Lactobacilli influence mRNA expression of type I IFN and inflammatory cytokines in PIE cells after poly(I:C) challenge

**Figure 2:**
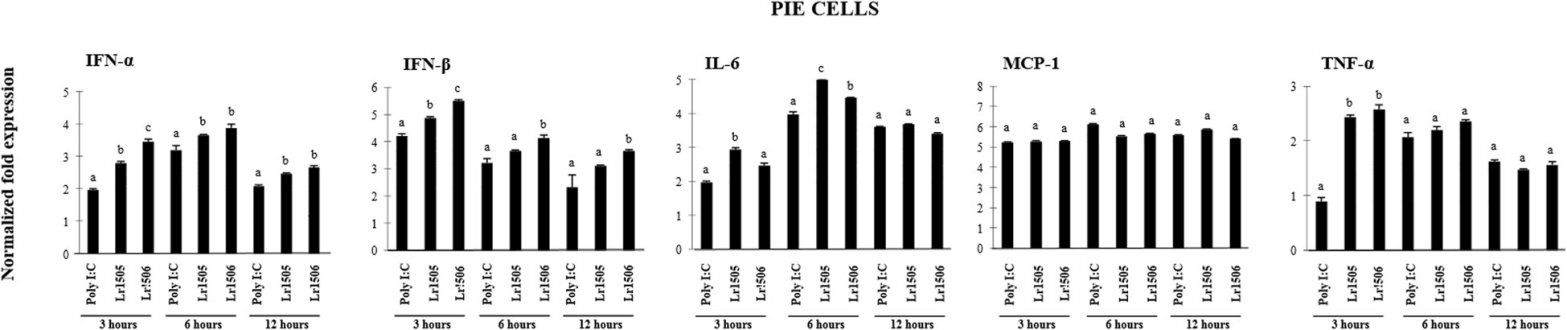
**Effect of immunobiotic lactobacilli in the response of porcine intestinal epithelial (PIE) cells to poly(I:C) challenge.** Monocultures of PIE cells were stimulated with *Lactobacillus rhamnosus* CRL1505 (Lr1505) or *L. rhamnosus* CRL1506 (Lr1506) for 48 hours and then challenged with poly(I:C). The mRNA expression of IFN-α, IFN-β, IL-6, MCP-1 and TNF-α was studied in PIE cells at different time points after challenge. Cytokine mRNA levels were calibrated by the swine β-actin level and normalized by common logarithmic transformation. Values represent means and error bars indicate the standard deviations. The results are means of 3 measures repeated 4 times with independent experiments. The mean differences among different superscripts letters were significant at the 5% level.

In order to investigate whether Lr1505 and Lr1506 were able to modify PIE cells response to a viral challenge, we used the dsRNA analogue poly(I:C) to stimulate PIE cells after pre-incubating them with the respective *Lactobacillus* strains. Interestingly, PIE cells reacted differently towards the single *L. rhamnosus* strains. Both Lr1505 and Lr1506 were able to significantly up-regulate the mRNA expression of IFN-α and IFN-β after poly(I:C) challenge. However, as depicted in Figure 2, while Lr1506 had a stronger effect on the production of type I interferons, Lr1505 had a higher influence on IL-6 mRNA expression. In addition, both strains equally increased the mRNA expression of TNF-α in poly(I:C)-challenged PIE cells while no significant effect was observed on the mRNA expression of MCP-1 at any time tested (Figure 2).

### Lactobacilli activate APCs and differentially modulate the expression of cytokines and activation markers in response to poly(I:C)

**Figure 3:**
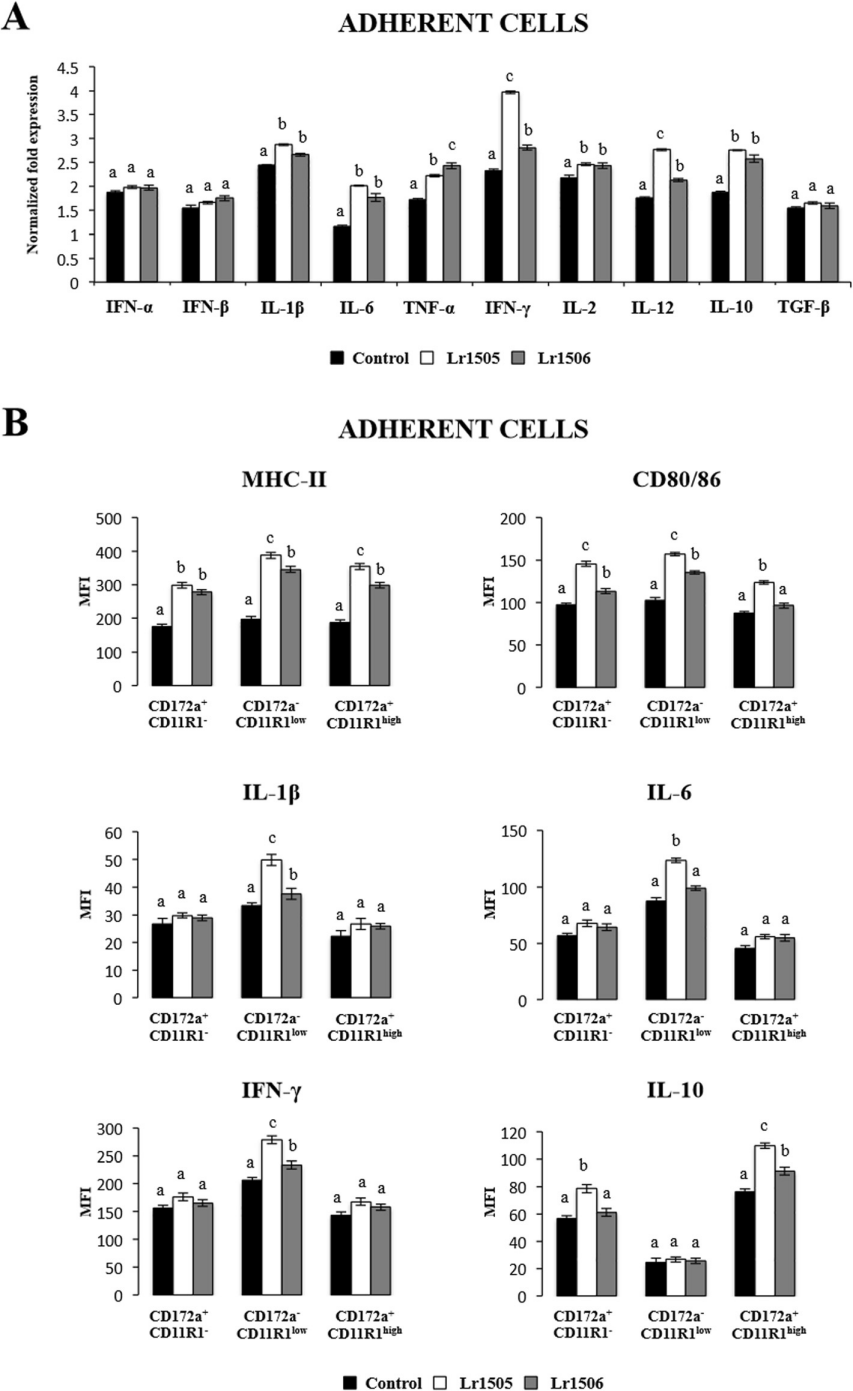
**Effect of immunobiotic lactobacilli in porcine antigen presenting cells (APCs) from Peyer’s patches. (A)** Monocultures of adherent cells from Peyer’s patches were stimulated with *Lactobacillus rhamnosus* CRL1505 (Lr1505) or *L. rhamnosus* CRL1506 (Lr1506) for 12 hours and then challenged with poly(I:C). The mRNA expression of IFN-α, IFN-β, IL-1β, TNF-α, IFN-γ, IL-6, IL-2, IL-12, IL-10 and TGF-β was studied after 12 hours of stimulation. Cytokine mRNA levels were calibrated by the swine β-actin level and normalized by common logarithmic transformation. **(B)** In addition, expression of MHC-II and CD80/86 molecules as well as intracellular levels of IL-1β, IL-10, IFN-γ and IL-10 were studied in the three populations of APCs within adherent cells defined with CD172a and CD11R1 markers. Values represent means and error bars indicate the standard deviations. The results are means of 3 measures repeated 4 times with independent experiments. The mean differences among different superscripts letters were significant at the 5% level.

We next evaluated the capacity of Lr1505 and Lr1506 to modulate the antiviral response triggered by poly(I:C) stimulation in adherent cells. Using this *in vitro* model, which mimics de context of intestinal viral infection we proved that lactobacilli not only modulated the response of PIE cells but also modulated several cytokines transcripts in immune adherent cells from PPs (Figure 3). As expected, poly(I:C) challenge induced an increase in the transcriptional levels of almost all cytokines tested in adherent cells. Lr1505 and Lr1506 exerted in general an improvement in the mRNA expression of cytokines in response to poly(I:C) challenge (Figure 3A). IL-1β, TNF-α, IFN-γ, IL-2, IL-12, and IL-10 mRNA levels were significantly higher in lactobacilli-treated cells than in controls while the mRNA expression of IFN-α, IFN-β and TGF-1β was not modified by Lr1505 or Lr1506 (Figure 3A). In addition, we observed that both strains were equally effective to improve mRNA expression of all the mentioned cytokines with the exception of IFN-γ and IL-12 which were significantly higher in Lr1505-treated cells when compared with those stimulated with Lr1506 (Figure 3A).

In parallel experiments using the same stimulation protocols, we studied the expression of surface activation markers and protein cytokine levels by flow cytometry in CD172a^+^CD11R1^−^, CD172a^−^CD11R1^low^ and CD172a^+^CD11R1^high^ adherent cells (Figure 3B). Challenge with poly(I:C) significantly increased the expression of surface molecules MHC-II and CD80/86 in the three populations of APCs. In addition, we observed that lactobacilli-treated cells showed higher levels of MHC-II and CD80/86 when compared to control cells with the exception of CD80/86 in Lr1506-treated CD172a^+^CD11R1^high^ cells that was similar to controls (Figure 3B). We also observed differences in the up-regulation of both molecules when comparing Lr1505 and Lr1506, since MCH-II levels in CD172a^−^CD11R1^low^ and CD172a^+^CD11R1^high^ adherent cells and CD80/86 levels in the three populations of APCs were higher in Lr1505-treated cells than in those stimulated with Lr1506 (Figure 3B). We also observed an up-regulation of IL-1β, IL-6, IL-10 and IFN-γ in poly(I:C) challenged APCs (Figure 3B) after being treated with *L. rhamnosus* strains. When studying the influence of lactobacilli on the distinct populations of APCs, we observed a differential behaviour towards each cell group. IL-1β, IL-6 and IFN-γ levels were significantly higher in lactobacilli-treated CD172a^−^CD11R1^low^ cells when compared to controls. Moreover, Lr1505 was more efficient than Lr1506 to up-regulate the levels of the three cytokines in that cell population (Figure 3B). On the other hand, IL-10 levels were significantly higher in lactobacilli-treated CD172a^+^CD11R1^−^ and CD172a^+^CD11R1^high^ cells when compared to controls. Moreover, Lr1505 was more efficient than Lr1506 to up-regulate the levels of IL-10 in both cell populations (Figure 3B).

### Lactobacilli differentially modulate cytokine expression in response to poly(I:C) challenge in APCs co-cultured with PIE cells

**Figure 4:**
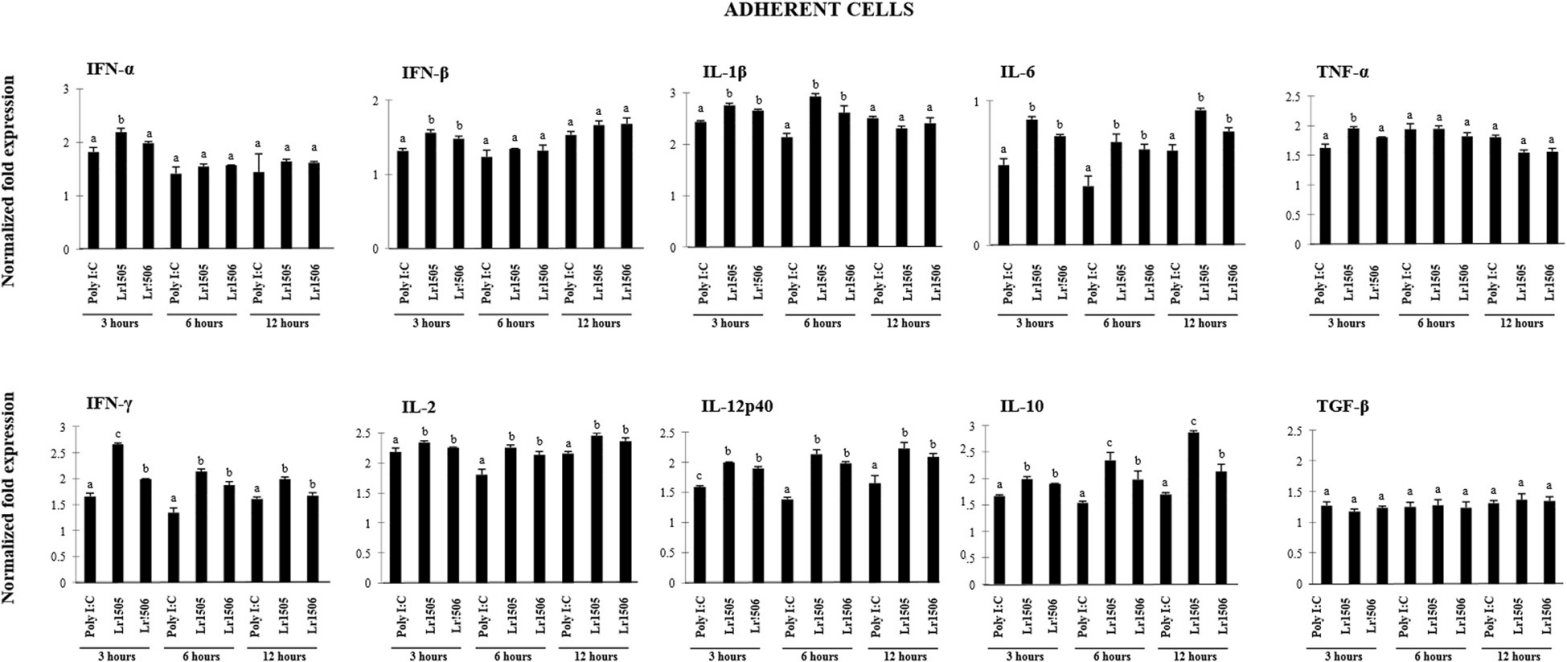
**Effect of immunobiotic lactobacilli in porcine antigen presenting cells (APCs) from Peyer’s patches co-cultured with porcine intestinal epithelial (PIE) cells.** PIE cells were co-cultured with adherent cells from Peyer’s patches and stimulated with *Lactobacillus rhamnosus* CRL1505 (Lr1505) or *L. rhamnosus* CRL1506 (Lr1506) for 12 hours. PIE-APCs co-cultures were then challenged with poly(I:C). The mRNA expression of IFN-α, IFN-β, IL-1β, TNF-α, IFN-γ, IL-6, IL-2, IL-12, IL-10 and TGF-β was studied at different time points after challenge. Cytokine mRNA levels were calibrated by the swine β-actin level and normalized by common logarithmic transformation. Values represent means and error bars indicate the standard deviations. The results are means of 3 measures repeated 4 times with independent experiments. The mean differences among different superscripts letters were significant at the 5% level.

Although the studies in PIE cells and adherent cells demonstrated the ability of Lr1505 and Lr1506 to modulate the response to poly(I:C) challenge, these *in vitro* models are simplified and may neglect the effect of cell–cell interactions in a complex organic microenvironment, which completely changes the resulting response. Then we used an *in vitro* PPs model culture system to evaluate the effect of both Lr1505 and Lr1506 more precisely. Co-cultures of PIE and adherent cells were treated with Lr1505 or Lr1506 and then stimulated with poly(I:C). mRNA expression of type I IFN and pro- and anti-inflammatory cytokines were measured at different times post-stimulation as shown in Figure 4. Changes induced by lactobacilli in PIE cells co-cultured with adherent cells were similar to those observed in PIE cells monocultures (data not shown). In adherent cells, poly(I:C) challenge increased the mRNA expression of INF-α, INF-β, and TNF-α and a significant increase was seen only in hour 3 in cells stimulated with Lr1505 whereas Lr1506 did not affected the mRNA expression of INF-α and TNF-α, and slightly influenced the IFN-β levels at this single time point (Figure 4). In addition, IL-1β, IFN-γ, IL-6, IL-2, and IL-12p40 were up-regulated by lactobacilli treatments (Figure 4). IFN-γ, IL-6, IL-2, and IL-12p40 up-regulation by both strains was sustained over time as it could be observed after 3, 6 and 12 hours post-poly(I:C) challenge and interestingly, levels of IFN-γ transcript in Lr1505-treated cells was significantly higher than those observed in Lr1506-treated cells at hour 3 (Figure 4). IL-10 was the only cytokine whose up-regulation increased gradually reaching a maximum level at hour 12 post-challenge. Lactobacilli-treated cells showed significantly higher levels of IL-10 mRNA expression however, Lr1505 showed a higher capacity to up-regulate IL-10 especially in the later time points studied (Figure 4). TGF-β mRNA expression suffered no changes at any time point tested (Figure 4). These results indicate that APCs can be indirectly modulated by both lactobacilli strains through their actions on IECs.

### TLR2 but not TLR9 would be involved in the immunoregulatory effect of lactobacilli

**Figure 5:**
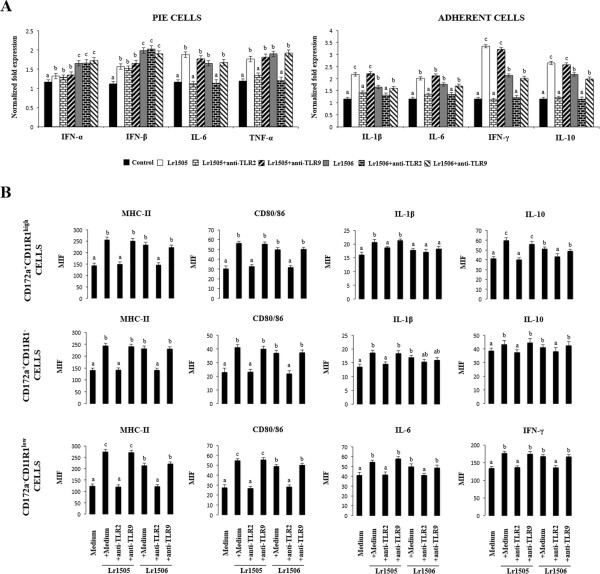
**Role of toll-like receptor (TLR)-2 and TLR9 in the immunoregulatory effect of immunobiotic lactobacilli in porcine intestinal epithelial (PIE) cells and antigen presenting cells (APCs) from Peyer’s patches.** Monocultures of PIE cells or adherent cells from Peyer’s patches were stimulated with *Lactobacillus rhamnosus* CRL1505 (Lr1505) or *L. rhamnosus* CRL1506 (Lr1506) with or without the addition of anti-TLR2 or anti-TLR9 blocking antibodies. The mRNA expression of IFN-α, IFN-β, IL-6, MCP-1 and TNF-α was studied in PIE cells after 48 hours of stimulation **(A)**. The mRNA expression of IFN-α, IFN-β, IL-1β, TNF-α, IFN-γ, IL-6, IL-2, IL-12, IL-10 and TGF-β was studied in adherent cells after 12 hours of stimulation **(A)**. Cytokine mRNA levels were calibrated by the swine β-actin level and normalized by common logarithmic transformation. In addition, expression of MHC-II and CD80/86 molecules as well as intracellular levels of IL-1β, IL-10, IFN-γ and IL-10 **(B)** were studied in the three populations of APCs within adherent cells defined with CD172a and CD11R1 markers. Values represent means and error bars indicate the standard deviations. The results are means of 3 measures repeated 4 times with independent experiments. The mean differences among different superscripts letters were significant at the 5% level.

We next aimed to evaluate whether TLR2 and/or TLR9 were involved in the immunomodulatory capacities of lactobacilli in PIE and adherent cells. Then, cells were stimulated again with Lr1505 or Lr1506 in the presence or absence of blocking anti-TLR2 or anti-TLR9 antibodies (Figure 5A). When analyzing cytokines transcripts in PIE cells, it was evident that neither TLR2 nor TLR9 were involved in the up-regulation of type I IFNs induced by Lr1505 and Lr1506. In contrast, in the presence of anti-TLR2 blocked the increase of IL-6 and TNF-α transcripts induced by Lr1505 and Lr1506 in PIE cells (Figure 5A). In addition, anti-TLR2 antibodies significantly blocked the increase of IL-1β, IL-6, IFN-γ, and IL-10 transcripts induced by Lr1505 and Lr1506 in PPs adherent cells while anti-TLR9 antibodies did not modified the immunomodulatory activities of lactobacilli (Figure 5A). We confirmed the involvement of TLR2 but not TLR9 in the activation of PPs adherent cells using flow cytometry. In CD172a^+^CD11R1^−^, CD172a^−^CD11R1^low^ and CD172a^+^CD11R1^high^ adherent cells the addition of anti-TLR2 significantly reduced the capacity of both Lr1505 and Lr1506 to up-regulate the expression of MHC-II, CD80/86, IL-1β, IL-6, IFN-γ, and IL-10 (Figure 5B).

**Figure 6:**
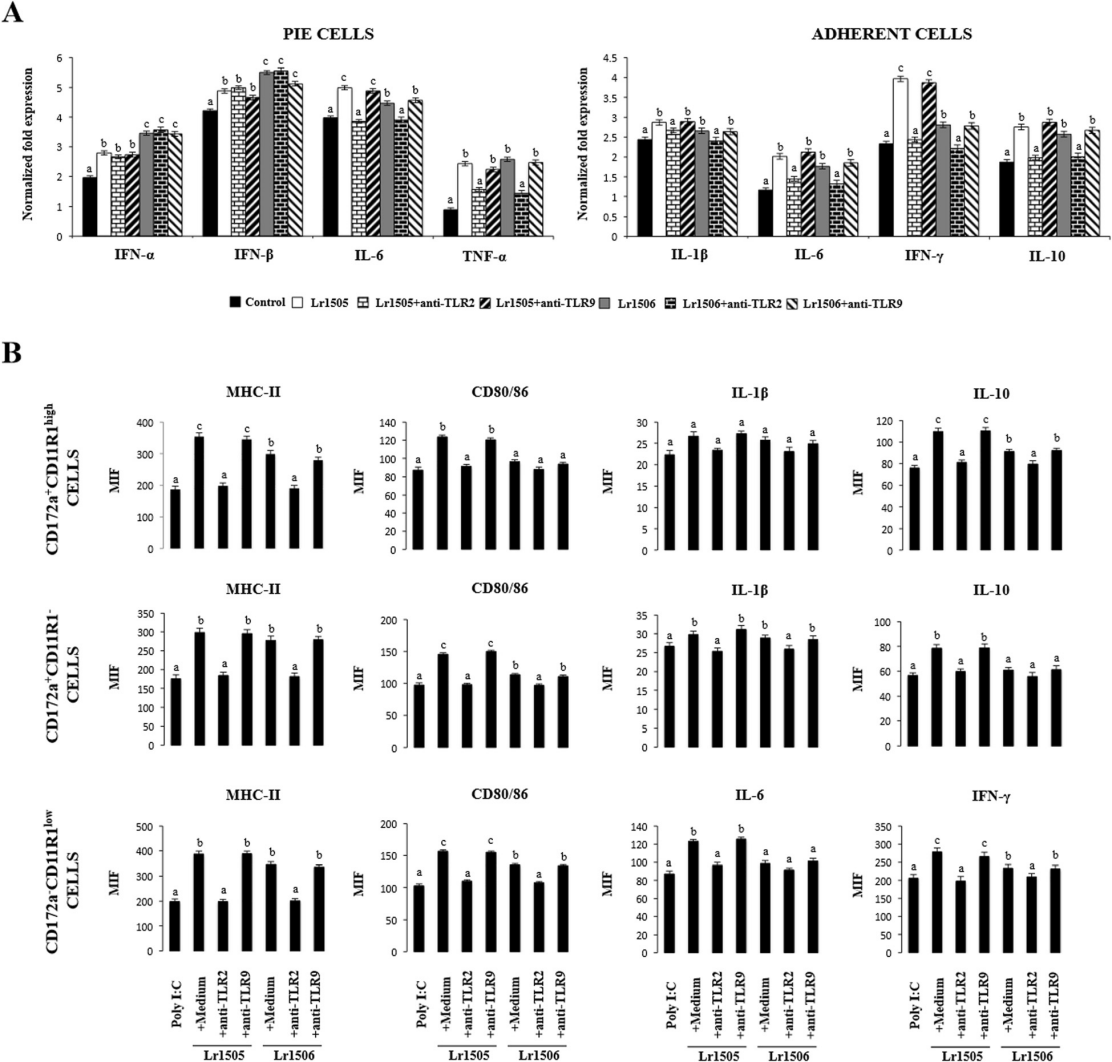
**Role of toll-like receptor (TLR)-2 and TLR9 in the immunoregulatory effect of immunobiotic lactobacilli in porcine intestinal epithelial (PIE) cells and antigen presenting cells (APCs) from Peyer’s patches in response to poly(I:C).** Monocultures of PIE cells or adherent cells from Peyer’s patches were stimulated with *Lactobacillus rhamnosus* CRL1505 (Lr1505) or *L. rhamnosus* CRL1506 (Lr1506) with or without the addition of anti-TLR2 or anti-TLR9 blocking antibodies. PIE and APCs were then challenged with poly(I:C). The mRNA expression of IFN-α, IFN-β, IL-6, MCP-1 and TNF-α in PIE and the mRNA expression of IFN-α, IFN-β, IL-1β, TNF-α, IFN-γ, IL-6, IL-2, IL-12, IL-10 and TGF-β in adherent cells was studied after 12 hours of poly(I:C) challenge **(A)**. Cytokine mRNA levels were calibrated by the swine β-actin level and normalized by common logarithmic transformation. In addition, expression of MHC-II and CD80/86 molecules as well as intracellular levels of IL-1β, IL-10, IFN-γ and IL-10 **(B)** were studied in the three populations of APCs within adherent cells defined with CD172a and CD11R1 markers. Values represent means and error bars indicate the standard deviations. The results are means of 3 measures repeated 4 times with independent experiments. The mean differences among different superscripts letters were significant at the 5% level.

Finally we evaluate the role of TLR2 and TLR9 in the modulation of the response against poly(I:C) challenge induced by lactobacilli (Figure 6A). Again, anti-TLR2 antibodies blocked the increase of IL-6 and TNF-α transcripts induced by Lr1505 and Lr1506 in PIE cells while no modification was observed for type I IFNs mRNA expression (Figure 6A). In a similar setting but using PPs APCs, the increase in transcriptional levels of IL-1β, IL-6, IFN-γ, and IL-10 in poly(I:C)-challenged adherent cells induced by lactobacilli was blocked when anti-TLR2 antibodies were present in the medium (Figure 6A). In addition, the ability of Lr1505 and Lr1506 to induce higher levels of MHCII and CD80/86 in poly(I:C)-challenged adherent cells was significantly blocked with anti-TLR2 antibodies (Figure 6B). Moreover, when studying the expression of IL-6, IFN-γ, IL-1β and IL-10 at post-translational levels in APCs stimulated with lactobacilli and then challenged with poly(I:C), MIF values remained at the same level of poly(I:C)-challenged control cells if the medium was added with anti-TLR2 antibodies (Figure 6B). In none of the experiments performed here, anti-TLR9 antibodies exerted any kind of effect on the expression of cytokines or molecules related to the antigen presenting process (Figure 6B).

## Discussion

Rotavirus represents one of the prevailing causes of infectious gastroenteritis in humans worldwide [3,4,6]. An initial and essential step in the viral infection cycle of rotavirus is entering and replicating in IECs of the small intestine [25]. IECs have been well defined as sentinels, because they are the first cells which encounter microorganisms and are not only a physical barrier but they recognize different types of PAMPs via PRRs, which are selectively expressed on the cell surface, internal compartments or cytoplasm. Upon virus internalization, dsRNA molecules are generated in infected cells [25]. These molecules are typical of many viral infections including rotavirus. Viral dsRNA activate PRRs such as TLR3, RIG-I, and MDA-5, which signal host cellular responses in order to try to control viral infection [25-27]. IFNs and IFN-regulated gene products are then synthesized and play a key role in the host response for clearing viruses. Type I together with type II IFNs are able to limit rotavirus infection *in vitro* and their levels are augmented in rotavirus-infected children and animals [18,28,29]. Recently, it has been proposed that IFNs signalling is not only beneficial to the host, but it may also enhance rotavirus replication at the first stages of infection [30]. Nevertheless, other *in vivo* studies have shown a markedly increase in the virulence of certain strains of rotavirus when IFNs signalling was blocked during infection [31]. Furthermore, the fact that rotavirus has evolved mechanisms to manipulate IFNs signalling such as the type I IFNs damping NSP1 protein [32], strongly suggests that IFNs are crucial to limit infection. Therefore, approaches aiming to modulate pathways leading to IFNs production may provide valuable tools to increase natural viral defence mechanisms. Herein we show evidence of how IECs can be modulated by immunobiotic *L. rhamnosus* in a strain-dependent fashion to enhance antiviral responses. For instance, Lr1506 was a stronger inducer of both IFN-α and IFN-β than Lr1505. In addition, these strains primed PIE cells to respond to the dsRNA analogue poly(I:C), as the cells responded with a significantly stronger synthesis of mRNA encoding for type I IFNs than non-treated cells. Moreover, the exposition of IECs to Lr1506 resulted in a significantly stronger up-modulation of type I IFNs mRNA expression than the treatment with Lr1505.

Although activation of PPRs signalling pathways, especially upon stimulation with their respective ligands have been extensively studied, research on the specific effect and modulation capability of probiotics including whole live LAB is more recent and in general includes different species of Gram-positive bacteria. We have reported previously, the modulation of type I IFNs in PIE cells by lactobacillus strains, specifically *Lactobacillus casei* MEP221106 [23]. Other studies on type I IFN induction and/or modulation by lactobacilli have only been reported for professional immune cells such as macrophages, DCs and PBMC but are rare for IECs. Furthermore, our results using blocking anti-TLR2 and anti-TLR9 antibodies ruled out the involvement of both TLR2 and TLR9 (the classical TLRs associated to LAB recognition) in the primary induction of type I IFNs or the enhancement of IFN-α and -β synthesis upon poly(I:C) challenge induced by Lr1505 and Lr1506 in PIE cells. Further studies are needed in order to find the PRRs involved in the recognition of lactobacilli leading to IFN-α and IFN-β expression in PIE cells.

IECs are able to initiate and in a minor extent to regulate the immune response to bacteria and viruses [33] being able to secrete several pro-inflammatory cytokines such as MCP-1, IL-6 and TNF-α on stimulation by pathogens. Both Lr1505 and Lr1506 were able to induce IL-6 and TNF-α mRNA expression in PIE cells but not MCP-1. This fact contrasts with a previous report about another probiotic strain, *L. casei* CRL431, which induces MCP-1 in murine IECs, which may be explained as both a strain-specific and/or a host-specific phenomenon [34]. In addition, not all IEC lines (e.g.: Caco-2, HT29, T84) are able to produce the same cytokine profile upon stimulation, and therefore, there are contradictory reports on the ability of lactobacilli and other Gram-positive commensal bacteria to induce IL-6 in IECs. Thus, as already suggested, this may be one advantage of working with IECs primary cultures [34]. Vinderola et al. [34] reported induction of IL-6 by probiotic lactobacilli in normal murine IECs as it was also the case for the effect on porcine IECs reported in this study. Our results using anti-TLR2 blocking antibodies proved that TLR2 is responsible for the recognition of lactobacilli and induction of IL-6 and TNF-α, which agrees with the results of Castillo et al. [35].

Dendritic cells are leading gatekeepers and regulators of immunity, which are present in all tissues, especially at the interface with the external environment, such as the mucosa of the gastrointestinal tract [36]. In the gut, they play a fundamental role as they orchestrate the subtle equilibrium between tolerance and protection against infection [37]. We and others have reported that probiotic lactobacilli are able to differentially stimulate and modulate DCs *in vitro*[22,23,37-40]. Thus, we wanted to study how the two immunobiotic *L. rhamnosus* strains reported here functionally modulate porcine PPs-derived adherent immune cells (CD172a^+^CD11R1^−^, CD172a^−^CD11R1^low^ and CD172a^+^CD11R1^high^ cells). The main effect of incubating *L. rhamnosus* with the single populations of immune adherent cells, resulted in differential mRNA expression of the key polarizing cytokines IL-1β, IL-6 and IFN-γ, which determine the fate of naïve T-cells. Lr1505 was the strain with the highest capacity to functionally modulate APCs. Considering CD172a^+^CD11R1^high^ and CD172a^−^CD11R1^low^ cells as DCs [21], and as such with the ability to favour Th1, Th2, Th17 or Treg immune responses, the increases in both IFN-γ and IL-12 induced especially by Lr1505, may lead to a Th1 response if we extrapolate this data to an *in vivo* situation. Furthermore, IFN-γ and IL-1β have been shown to have a direct effect on IECs inducing an antiviral program, which inhibits rotavirus entry [41,42].

On the other hand, Lr1505 also induced IL-10 mRNA and protein expression, which is an immunoregulatory cytokine that avoids inflammatory-tissue injury during infections. Zhou et al. [43] provided direct evidence that aberrant activation of intestinal immunity induced by poly(I:C) or purified rotavirus genomic dsRNA causes a breakdown of the mucosal homeostasis, leading to mucosal damage. Moreover, it was reported that the induction of the regulatory IL-10 plays an important role to control the inflammatory process upon a viral infection to minimize tissue injury [39,44]. Then, the improved production of IL-10 induced by Lr1505 in APCs after the challenge with poly(I:C) could have an important protective effect during intestinal viral infections.

It is well known that commensal microbiota interacts with cells of the intestinal mucosa via TLR [36] but not all bacteria have the ability to modulate immune responses, as this is a strain specific characteristic. As lactobacilli may be recognized by APCs through the peptidoglycan and lipoteichoic acid in their cell walls and/or CpG motifs in their DNA, we used anti-TLR2 and anti-TLR9 antibodies to block recognition via the respective receptors in order to elucidate whether they were responsible for the observed immunoregulatory activity of lactobacilli in APCs. TLR2 is one of the PRRs that would be of great importance for the immunomodulatory effect of probiotic microorganisms in APCs. Immunoenhancing lactobacilli are able to increase the expression of TLR2 in DCs and macrophages isolated from PPs in mice [45] and in human myeloid DCs [46]. Moreover, Weiss et al. [40] reported a TLR2-dependent mechanism for *L. acidophilus* NCFM, whose IFN-β expression was markedly reduced in TLR-2^−/−^ DCs. In our experiments, the main effect observed on type I IFNs was observed in PIE cells and not in immune cells. After the challenge of APCs with poly(I:C), we observed a weak enhancement of type I IFNs mRNA expression, which was only 3 h after stimulation and therefore was not further studied. On the contrary, we observed a clear involvement of TLR2 signalling pathway in the up-modulation of IL-1β, IL-6, IL-10 and IFN-γ in APCs exerted by both *L. rhamnosus* strains alone and following a poly(I:C) challenge. In addition, the lactobacilli reported by Plantinga et al. [47] induced cytokines in DCs in a TLR9-dependent manner, contrasting our results which show no relationship between TLR9 and the immunoregulatory effect of Lr1505 or Lr1506.

## Conclusions

**Figure 7:**
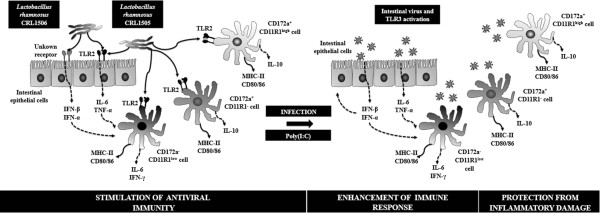
**Proposed mechanism for the immunoregulatory effect and antiviral activities of *Lactobacillus rhamnosus *** **CRL1505 and *L. rhamnosus *** **CRL1506 on porcine intestinal epithelial cells and antigen-presenting cells from swine Peyer’s patches.**

There is a general concept that the overall effect of probiotics is strain-specific, but there are only a few comparative studies where at least two strains of the same species provide significant differences in their immunomodulatory potential [38]. Herein, we show that two strains, both *L. rhamnosus*, isolated from the same ecological niche and with similar technological properties [10,11], are capable to induce differential antiviral defence phenotypes in IECs and APCs. We propose a model of action for each strain as depicted in Figure 7. In general terms, Lr1506 has a marked influence on IECs and antiviral innate defence mediated by type I IFNs, whereas Lr1505 stands out for its influence on APCs.

Both Lr1505 and Lr1506 were able to induce IFN–α and –β mRNA expression in IECs and improve the production of type I IFNs in response to poly(I:C) challenge independently of TLR2 or TLR9 signalling. However, Lr1506 showed a higher capacity to improve levels of IFN-α and IFN-β in IECs when compared with Lr1505, which is in line with our previously reported *in vivo* results, showing higher levels of IFN-α and IFN-β in intestinal fluids of Lr1506-treated than in Lr1505-treated mice [16]. Considering that type I IFNs up-regulate several genes involved in viral defence and genes of major importance for the development of a strong cellular response, we hypothesize that Lr1506 may play an important role in the improvement of innate immune responses against intestinal virus, especially in IECs.

In addition, both lactobacilli induced expression of IL-6 and TNF-α via TLR2 in IECs, being Lr1505 the stronger modulator of these cytokines. Furthermore, although both strains were able to significantly increase surface molecules expression and cytokine production in intestinal APCs, Lr1505 had a stronger effect both when applied alone or combined with a posterior poly(I:C) challenge. The improved Th1 response induced by Lr1505 was triggered by TLR2 signalling and included augmented expression of MHC-II and co-stimulatory molecules and expression of IL-1β, IL-6, and IFN-γ in APCs (Figure 7). Considering that TLR signalling is a crucial aspect of innate defence [48,49], but if uncontrolled at mucosal surfaces, it would be pathological, it is important to highlight again the fact that IL-10 was also significantly up-regulated by Lr1505, suggesting that the inflammatory conditions may be held under control (Figure 7). These *in vitro* results are in line with our previous findings showing that Lr1505 was more efficient than Lr1506 for increasing the levels of IFN-γ, IL-10 and IL-6 in the intestine of mice [16].

It was recently reviewed the emergence of TLR agonists as new ways to transform antiviral treatments by introducing panviral therapeutics with less adverse effects than IFN therapies [50]. The use of *L. rhamnosus* CRL1505 and *L. rhamnosus* CRL1506 as modulators of innate immunity and inductors of antiviral type I IFNs, IFN-γ, and regulatory IL-10 clearly offers the potential to overcome this challenge. To evaluate *in vitro* and *in vivo* the capacity of both strains to protect against rotavirus infection is an interesting topic for future research.

## Competing interests

The authors declare that they have no competing interests.

## Authors’ contributions

JV, YT, SA and HK conceived the study; JV, EC, YT, HI, SA and HK designed the study; JV, EC, MGV, YT, TT, TI and SS did the laboratory work. JV, EC, MGV, YT, TT, TI, SS, SA and HK analysed the data. JV, MGV and HK wrote the manuscript; all read and approved the manuscript.
